# Reply to Hinpetch Daungsupawong et al.

**DOI:** 10.1093/icvts/ivag153

**Published:** 2026-05-24

**Authors:** Shunsuke Matsushima, Hironori Matsuhisa, Kenji Okada, Osamu Kawanami

**Affiliations:** Department of Cardiovascular Surgery, Kobe Children’s Hospital, Kobe 650-0047, Japan; Division of Cardiovascular Surgery, Department of Surgery, Kobe University Graduate School of Medicine, Kobe 650-0017, Japan; Department of Cardiovascular Surgery, Kobe Children’s Hospital, Kobe 650-0047, Japan; Division of Cardiovascular Surgery, Department of Surgery, Kobe University Graduate School of Medicine, Kobe 650-0017, Japan; Department of Mechanical Engineering, University of Hyogo, Himeji 671-2280, Japan; Advanced Medical Engineering Research Institute, University of Hyogo, Himeji 670-0836, Japan

We thank Daungsupawong and Wiwanitkit for their insightful comments on our article titled “In Vitro Hydrodynamic Evaluation of Pulmonary Expanded Polytetrafluoroethylene Valved Conduits.”^1^^,^^2^ Their constructive criticism mentioned the gap between our in vitro setup and actual clinical situations and the limited number of experimental conditions and subjects. They also raised questions about the predictive value of hydrodynamic characterization for conduit durability. These valid points do not only reaffirm our study limitation but also provide an opportunity to highlight our inventive attempt to identify functional consequences of conduit design differences.

Clinical data of pulmonary-valved conduits in the existing literature is extremely heterogeneous. In addition to conduit type, multiple factors such as patient background, conduit size, and surgical technique influence the outcomes and obscure the detail of each conduit failure. Its true elucidation would require a prospective randomized controlled trial, which is impractical as well as ethically infeasible. Therefore, experimental systems that allow each factor to be unravelled one by one are needed. In this study, circulatory condition and conduit size were fixed as a representative condition to evaluate the effect of root design differences. We believe that this investigation serves as one step toward a deeper understanding of pulmonary valved conduits and expect that future multi-condition experiments with various conduits will contribute to the advancement of this field.

Furthermore, our methodology is specifically focused on comparing the engineering performance of the valves. Hydrodynamic metrics derived from particle image velocimetry can objectively examine the intrinsic merits and drawbacks of each design. For the development of better valve products, these design requirements should also be met while considering biological and material issues. As there may be a logical leap in linking hydrodynamic metrics and valve longevity, our conclusion regarding conduit durability remains speculative. However, it appears to be reasonable that excessive shear stress around the conduit should induce persistent subclinical inflammation and thrombosis and cause premature structural valve deterioration. This is a challenge that seeks to anticipate both the immediate and decades-later implications of conduit implantation.
